# Luxations carpo-métacarpiennes dorsales du quatrième et cinquième métacarpiens sans fracture

**DOI:** 10.11604/pamj.2016.23.92.8369

**Published:** 2016-03-15

**Authors:** Bah Aliou, El Alaoui Adi

**Affiliations:** 1 Service de Chirurgie Orthopédique et Traumatologique, Centre Hospitalier Métropole Savoie de Chambéry, Chambéry, France; 2 Chirurgie Traumato-Orthopédie au Centre Hospitalier Universitaire de Fez, Fez, Maroc

**Keywords:** Luxation carpo-métacarpienne, dorsale, traumatisme métacarpien, Dorsal carpometacarpal dislocation, carpometacarpal trauma, orthopedic reduction

## Abstract

The frequency of the interesting carpometacarpal dislocation spacing is 1.93% of all injuries of the wrist and carpus. The first observations would return to Cooper and Roux in the nineteenth century. Traumatic carpometacarpal dislocations without fractures are rare. If left untreated, these lesions can lead to joint instability and early joint degeneration. We report a case of a dorsal carpometacarpal dislocation of and fourth and fifth metacarpals in a young man of 46 years, right-handed, security officer, who after a sudden nervousness lends a hand against the wall resulting in a total pain and functional impairment of his right hand. The clinical examination and radiological assessment (standard radiography and CT scan) confirm the diagnosis. We reduce its emergency dislocation by simply pulling on the axis with direct manual pressure on the bases of the metacarpals. His hand was immobilized for 6 weeks followed by rehabilitation. At 1 year follow-up, he showed no pain and returned all its activities without any discomfort.

## Image en médecine

La fréquence des luxations intéressant l'interligne carpo-métacarpien serait de 1,93% de l'ensemble des traumatismes du poignet et du carpe. Les premières observations reviendraient à Cooper et Roux au XIX^e^ siècle. Les luxations carpo-métacarpiennes traumatiques pures sans fracture associée sont des lésions rares. Non traitées, ces lésions peuvent mener à une instabilité articulaire et à une dégénérescence articulaire précoce. Nous rapportons le cas d'une luxation carpo-métacarpienne dorsale quatrième et cinquième métacarpiens chez un jeune homme de 46 ans, droitier, agent de sécurité, qui à la suite d'un coup d’énervement donne un coup poing contre le mur (mécanisme indirect) entrainant une douleur et impotence fonctionnelle totale de la main droite. L'examen clinique et le bilan radiologique (radiographie standard et Scanner) retiennent le diagnostic d'une luxation carpo-métacarpienne dorsale sans fracture de la quatrième et cinquième métacarpienne droite. Le traitement a consisté en une réduction, en urgence, de la luxation à foyer fermé par simple traction dans l'axe complétée par une pression manuelle directe sur les bases des métacarpiens. L'articulation a été immobilisée durant 6 semaines puis d'une rééducation de sa main droite. À 1 an de recul, il ne présentait aucune douleur et avait repris toutes ses activités sans aucune gên

**Figure 1:**
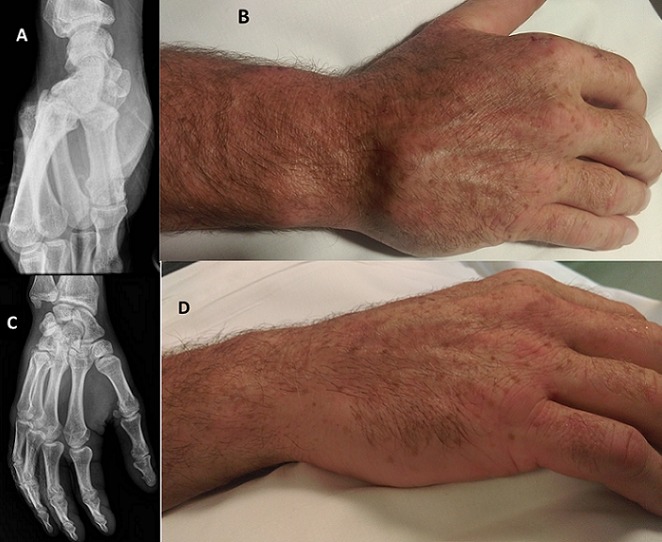
(A) radiographie standard de la main droite montrant la luxation carpo-métacarpienne du quatrième et cinquième métacarpiens; (B) la main droite montrant la luxation clinique carpo-métacarpienne du quatrième et cinquième métacarpiens; (C) radiographie standard de la main droite après réduction de la luxation carpo-métacarpienne du quatrième et cinquième métacarpiens; (D) la main droite après réduction

